# Possibility of Superconductivity of ^6^Li^+^ Ions in Solid Electrolytes at Room Temperature Under Coherent Acoustic Phonons

**DOI:** 10.3390/ma18133058

**Published:** 2025-06-27

**Authors:** Kyuichi Yasui

**Affiliations:** National Institute of Advanced Industrial Science and Technology (AIST), Nagoya 463-8560, Japan; k.yasui@aist.go.jp

**Keywords:** superconductivity, ^6^Li^+^ ions, boson, superfluidity, de Broglie wavelength, coherent acoustic phonons, thermal noise

## Abstract

It has been theoretically suggested that the de Broglie wavelength of Li ions could become longer than the Li atomic distance in solid electrolytes under coherent acoustic phonons at room temperature when thermal noise is sufficiently suppressed by them. This suggests that some quantum effect of Li ions (not electrons) could appear under this condition, which could possibly result in the superconductivity of ^6^Li^+^ ions (bosons) in solid electrolytes at room temperature. A lower frequency of coherent phonons is better for this possibility. A mechanism for the generation of coherent phonons by repetitive pulsed-laser irradiation or possibly by ultrasound irradiation using a transducer is also discussed.

## 1. Introduction

Conventional Li-ion batteries have a safety problem because the liquid electrolytes used in Li-ion batteries are flammable upon heating due to their vaporization [1,2]. To solve the safety problem, solid electrolytes have been intensively studied because they are not flammable without any leakage [3]. However, solid electrolytes usually have lower ionic conductivity compared to liquid electrolytes, which prevents higher power levels in all-solid-state batteries [2,4,5]. The authors of [6,7,8,9] have suggested that solid electrolytes filled with appropriate dislocations called all-dislocation-ceramics could have several orders of magnitude higher ionic conductivity than those of normal solid electrolytes, typically in the order of $10^{-3}$ S ${cm}^{-1}$ or less at room temperature.

What is the upper limit of ionic conductivity in solid electrolytes? It has been known that there are several solid electrolytes with extremely high ionic conductivity in the order of S ${cm}^{-1}$ at room temperature called superionic conductors such as $Rb{Ag}_{4}I_{5}$ with a ${Ag}^{+}$ conductivity of 0.25 S ${cm}^{-1}$ [10,11]. The maximum ionic conductivities of a solid ${Li}^{+}$ or ${Na}^{+}$ electrolyte are about $3.2\times 10^{-2}$ S ${cm}^{-1}$ and $4.1\times 10^{-2}$ S ${cm}^{-1}$, respectively [11,12,13]. The mechanism for the conventional superionic conduction is mostly due to a reduction in the energy barrier for ion motion by phonon–ion interaction [11,14,15,16]. There are also other models for superionic conduction, such as the existence of a free-ion state like a free-electron state in metals [17]. In the present paper, on the other hand, the possibility of purely quantum mechanical superconductivity is discussed, which may result in nearly infinite ionic conductivity.

Usually, superconductivity is known for electrons, especially at low temperatures [18,19,20]. After the discovery of high-$T_{c}$ superconductivity in the Ba-La-Cu-O system by Bednorz and Müller in 1986 [21], the onset temperature ($T_{c}$) of the superconductivity of electrons above the temperature of liquid nitrogen (77 K) was realized [22]. Now, the highest $T_{c}$ is 166 K for $Hg{Ba}_{2}{Ca}_{n-1}{Cu}_{n}O_{y}$ under 23 GPa [23,24]. For hydrogen-rich materials, $T_{c}$ near room temperature has been achieved under very high pressures; $T_{c}\approx 250$ K for $LaH_{10}$ under 180–200 GPa [25,26]. It is known that Cooper pairs of electrons are formed in superconductors, and the Bose–Einstein condensation of the bosons (Cooper pairs of electrons) corresponds to superconductivity, like the superfluidity of liquid helium [20,27]. The mechanism of Cooper pair formation is electron–phonon interaction according to the BCS theory [19,20]. For high-$T_{c}$ superconductivity, this could be due to direct electron–electron interactions, such as through spin fluctuations.

The condition of Bose–Einstein condensation is crudely expressed by the thermal de Broglie wavelength ($\lambda_{T}$) of the particle being larger than the mean distance (l) between the particles as follows, which is the condition of the appearance of the quantum effect due to the wave nature of the particles:

(1) $$\lambda_{T}\geq l={\left(\frac{V}{N}\right)}^{\frac{1}{3}}$$

where N is the number of particles in the volume V. The thermal de Broglie wavelength ($\lambda_{T}$) is given as follows [28]:

(2) $$\lambda_{T}=\frac{h}{\sqrt{2\pi mk_{B}T}}$$

where h is the Planck constant (=$6.626\times 10^{-34}$ J·s), m is the mass of a particle, $k_{B}$ is the Boltzmann constant (=$1.38\times 10^{-23}$ $JK^{-1}$), and T is the temperature in K. From Equations (1) and (2), the following condition is obtained:

(3) $$T\leq \frac{h^{2}}{2\pi mk_{B}}{\left(\frac{N}{V}\right)}^{\frac{2}{3}}$$

**Figure 1:**
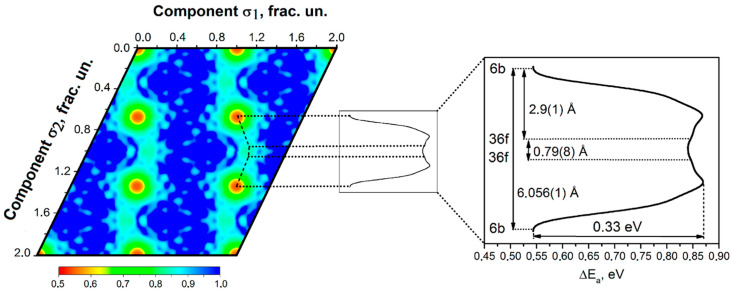
Two-dimensional section cut of the lithium one-particle potential and its 1D section along a straight chain of Li atoms in ${Li}_{1.3}{Al}_{0.3}{Ti}_{1.7}{\left(PO_{4}\right)}_{3}$ (LATP) solid electrolytes. Reprinted with permission from Ref. [32]. Copyright 2016, American Chemical Society.

However, the exact expression for the critical temperature ($T_{0}$) is given as follows [29]:

(4) $$T_{0}=\frac{3.31\hbar^{2}}{g^{2/3}mk_{B}}{\left(\frac{N}{V}\right)}^{\frac{2}{3}}$$

where ℏ=h/2π is the reduced Planck constant, and g is degeneracy (g=2S+1, where S is the spin of the particle). In any case, a crude estimate of the condition of Bose–Einstein condensation is given by Equations (1) and (2). This condition is, however, only for bosons. For fermions, Cooper pairs of fermions need to be formed for the Bose–Einstein condensation to occur. The temperature for the Cooper pair formation is considerably lower than the critical temperature ($T_{0}$) for the Bose–Einstein condensation given by Equation (4). For example, the critical temperature for the superfluidity of liquid ^3^He (fermion) is $2\times 10^{-3}$ K, which is significantly lower than that for liquid ^4^He (boson) of 2.17 K [27]. ${Li}_{1.3}{Al}_{0.3}{Ti}_{1.7}{\left(PO_{4}\right)}_{3}$ (LATP) is one of the promising solid electrolytes of a ${Li}^{+}$ ion conductor because of its structural stability, compatibility with high-voltage cathodes, and high ionic conductivity at room temperature [30,31]. The distance between ${Li}^{+}$ ions is about 6 Å=0.6 nm, as shown in Figure 1 [32]. Thus, in the present study, the mean distance (l) between ${Li}^{+}$ ions in solid electrolytes is assumed to be 0.6 nm.

Natural Li atoms consist of 92.4% ^7^Li (with an atomic mass of 7.0 u) and 7.6% ^6^Li (with an atomic mass of 6.0 u) in molar fraction. The nuclear spin of ^7^Li and ^6^Li is 3/2 and 1, respectively. In other words, the nucleus of ^7^Li and ^6^Li is a fermion and a boson, respectively. As the ${Li}^{+}$ ion has two electrons, ^7^Li^+^ and ^6^Li^+^ are fermions and bosons, respectively. Although ^6^Li^+^ could result in Bose–Einstein condensation as single particles, ^7^Li^+^ needs to become a boson by forming Cooper pairs.

According to Equation (4), the critical temperature is $T_{0}\approx 0.4$ K for the Bose–Einstein condensation of ^6^Li^+^ when $l={\left(V/N\right)}^{1/3}\approx 0.6$ nm. In other words, the superconductivity of the ionic conduction of ${Li}^{+}$ in solid electrolytes is impossible at room temperature because the thermal de Broglie wavelength of ${Li}^{+}$ ions is as small as 0.04 nm according to Equation (2) (it should be noted that proton superconductivity has been theoretically predicted in extremely high-density neutron stars [33,34]). In the experiment of the superconductivity of lithium metal below 0.4 mK at ambient pressure reported by Tuoriniemi et al. [35], some quantum effects of Li atoms could have occurred.

The de Broglie wavelength ($\lambda_{dB})$ is given as follows [36]:

(5) $$\lambda_{dB}=\frac{h}{\left|p\right|}$$

where p is the momentum of the particle. The thermal de Broglie wavelength at room temperature is relatively short due to the relatively large momentum of a particle owing to its thermal motion. It has been suggested, however, that thermal noise as well as quantum noise could be suppressed in coherent phonons [37,38,39,40]. With regard to the uncertainty principle (∆x∆p≥ℏ/2, where ∆x and ∆p denote uncertainty in position and momentum, respectively), coherent phonons are spatially spread out (∆x≈∞), and the resultant uncertainty in momentum could be nearly zero (∆p≈0). Coherent phonons are often excited by repetitive pulsed-laser irradiation with phonon frequencies ranging from GHz to THz [41]. For example, a femtosecond laser with a pulse width of 200 fs, a photon energy of 2 eV (corresponding to a 480 THz electromagnetic wave), and an energy per pulse of 0.2 nJ was used to generate phonons at around 25 GHz in semiconductors [42]. Coherent acoustic phonons may also be produced using piezoelectric oscillators [41]. Misochko [37] showed that thermal noise could be reduced when coherent phonons are excited by a very short laser pulse (femtosecond laser pulse) because the energy exchange of the system with the reservoir is markedly smaller than $k_{B}T$ during such a short period of time. Ventura-Velázquez et al. [38] showed by numerical simulations that thermal noise could be reduced in coherent phonons. Garret et al. [40] experimentally claimed to detect squeezed phonons. In the present paper, coherent longitudinal acoustic phonons are considered because a lower frequency of phonons is more suitable for reducing the magnitude of momentum in Equation (5) and increasing the de Broglie wavelength. Numerical calculations are performed to study conditions of the Bose–Einstein condensation (or superconductivity) of ^6^Li^+^ ions (bosons) in solid electrolytes under coherent acoustic phonons which could suppress thermal noise and increase de Broglie wavelengths at room temperature.

## 2. Model

For a relatively low frequency of acoustic phonons, the following dispersion relationship holds [43]:

(6) ω=ck

where ω is the angular frequency of an acoustic phonon (ω=2πf, where f is the frequency of an acoustic phonon), c is the sound velocity (c=6900 m/s for LATP [9]), and k is the wave number (k=2π/λ, where λ is the wavelength of the acoustic phonon). The displacement (y) of atoms (${Li}^{+}$ ions) in the direction of coherent-longitudinal-phonon propagation is expressed as follows:

(7) $$y=A\sin\left(kx-\omega t\right)$$

where A is the amplitude of oscillation, x is the position in the direction of the phonon propagation, and t is time. Accordingly, the velocity (v) of atoms (${Li}^{+}$ ions) is expressed as follows:

(8) $$v=\frac{\partial y}{\partial t}=-A\omega \cos\left(kx-\omega t\right)$$

As the associated momentum of atoms (^6^Li^+^ ions) is $p_{acoustic}=mv$, where m is the mass of a ^6^Li^+^ ion, the instantaneous de Broglie wavelength is given as follows from Equation (5):

(9) $$\lambda_{dB}=\frac{h}{\left(\left|p_{acoustic}\right|+C_{th}p_{th}\right)}$$

where $C_{th}$ is a fraction of the thermal noise that appeared in coherent acoustic phonons ($0\leq C_{th}\leq 1$), and $p_{th}$ is a component of the momentum of atoms (^6^Li^+^ ions) due to full thermal noise ($p_{th}=\sqrt{2\pi mk_{B}T}$ according to Equations (2) and (5)). Equation (9) is valid even in the crystal field of solid electrolytes (for example, Equation (5) is valid even inside a hydrogen atom for an electron [44]).

As already noted, the typical frequency of coherent acoustic phonons is GHz-THz [41]. Thus, in the present paper, numerical calculations are performed for 1 GHz (=$10^{9}$ Hz) and 1 THz (=$10^{12}$ Hz). For the amplitude (A) of oscillation in Equation (7), it typically ranges from 1 to 100 pm [45]. Accordingly, in the present paper, the results of numerical calculations with A=10 pm (=$10^{-11}$ m) are shown both for 1 GHz and 1 THz at room temperature (T=298.15 K). In the calculations of de Broglie wavelengths in Equation (9), the mass of ^6^Li^+^ is used ($m=6.0\times 1.66\times 10^{-27}$ kg) because the ^6^Li^+^ ion is a boson. However, the results for the ^7^Li^+^ ion are similar.

## 3. Results and Discussions

**Figure 2:**
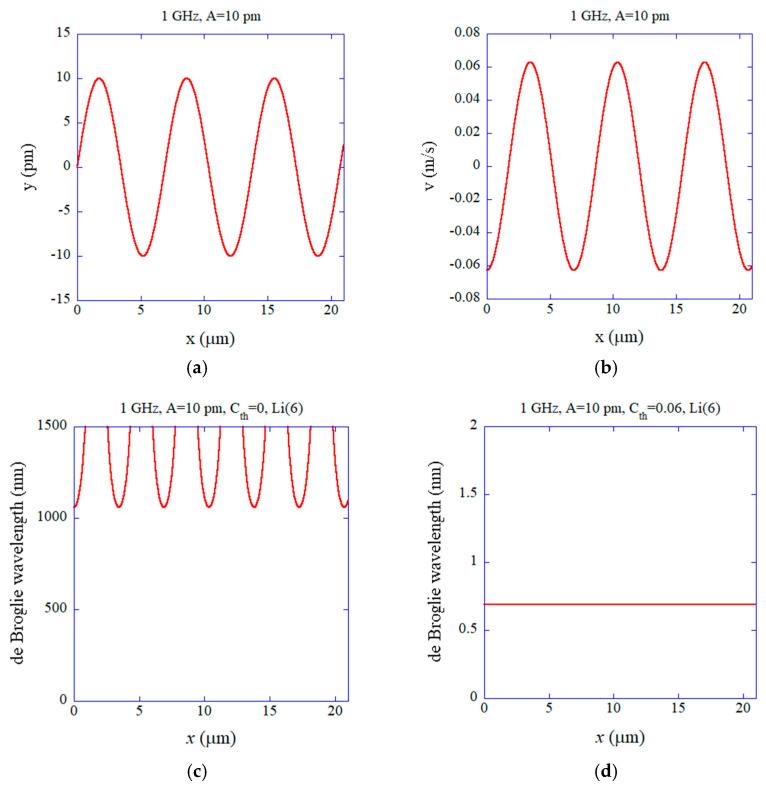
The results of numerical calculations as a function of position (*x*) along the direction of phonon propagation at *t* = 0 for coherent acoustic phonons of 1 GHz and at *A* = 10 pm. (**a**) The instantaneous displacement (*y*) of ^6^Li^+^ ions in the direction (x) of longitudinal-acoustic-phonon propagation. (**b**) The instantaneous velocity (*v*). (**c**) The instantaneous de Broglie wavelength of the ^6^Li^+^ ion without any thermal noise ($C_{th}=0$). (**d**) The instantaneous de Broglie wavelength for $C_{th}=0.06$.

In Figure 2, the results of the numerical calculations under 1 GHz and A=10 pm are shown as a function of position (x) at time t=0. The wavelength of the coherent acoustic phonons under the condition is λ=6.9 μm, and the horizontal axis of Figure 2 is for three wavelengths (3λ≈21 μm). The displacement (y) and velocity (v) of the ${Li}^{+}$ ions are shown in Figure 2a,b, respectively. The maximum amplitude of velocity is 0.0628 m/s in this case, which is relatively small. Accordingly, when the thermal noise is completely suppressed in the coherent acoustic phonons ($C_{th}=0$), the de Broglie wavelength of the ${Li}^{+}$ ion is always more than three orders of magnitude longer than the Li atomic distance (l=0.6 nm) (Figure 2c). This strongly suggests that some quantum effect of ${Li}^{+}$ ions appears in solid electrolytes under the condition. In other words, the superconductivity (superfluidity) of ^6^Li^+^ ions may be possible under these conditions. It has been pointed out, however, that an appreciable number of vacancies need to be present for the superfluidity of a solid to occur, which is called supersolids [27,46,47,48,49]. In the presence of vacancies, the frequency (ω) of coherent phonons may be changed [50]. Furthermore, the distance between neighboring ^6^Li^+^ ions could increase around a vacancy, which could reduce the fraction of the solid undergoing superconductivity. In the present study, all ${Li}^{+}$ ions in LATP are assumed to be ^6^Li^+^ ions (bosons). In the case of ^7^Li^+^ ions (fermions), Cooper pairs need to be formed for superconductivity (superfluidity) to occur, which may be possible through the interaction of ^7^Li^+^ ions and the electron cloud in solid electrolytes or direct ^7^Li^+^ ion–ion interaction. Further studies are required on the Cooper pair formation of ^7^Li^+^ ions in solid electrolytes under coherent acoustic phonons. In the case of a mixture of ^6^Li^+^ and ^7^Li^+^ ions in solid electrolytes, only a fraction of the solid undergoes superconductivity [27]. However, it may still be possible for superconductivity (superfluidity) to occur for the whole solid if Cooper pairs of ^7^Li^+^ ions are formed. Indeed, the superfluidity of a mixture of fermionic ^6^Li and bosonic ^7^Li atoms has been experimentally reported at a very low temperature below 130 nK [51].

When the thermal noise is suppressed to less than about 7% ($C_{th}\leq 0.07$), the de Broglie wavelength is still longer than the mean atomic distance of 0.6 nm (Figure 2d). Accordingly, under the condition ($C_{th}\leq 0.07$), the superconductivity of ^6^Li^+^ ions (bosons) may possibly occur in solid electrolytes at room temperature.

**Figure 3:**
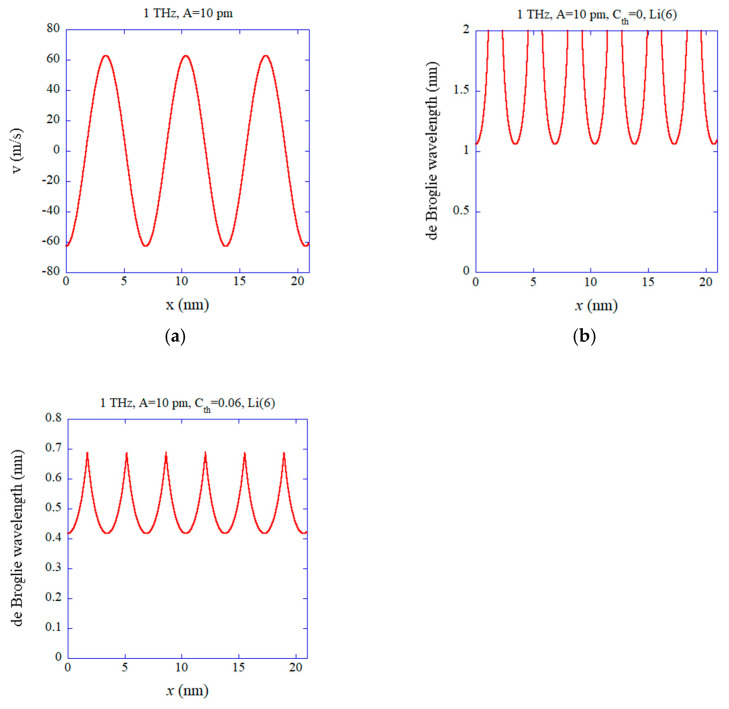
The results of numerical calculations for 1 THz. The other conditions are the same as those in Figure 2. (**a**) The instantaneous velocity (*v*). (**b**) The instantaneous de Broglie wavelength of the ^6^Li^+^ ion without any thermal noise ($C_{th}=0$). (**c**) The instantaneous de Broglie wavelength for $C_{th}=0.06$.

In Figure 3, the results of numerical calculations under 1 THz are shown. The other conditions are the same as those in Figure 2. The wavelength of coherent acoustic phonons is λ=6.9 nm in this case, and the horizontal axis is for three wavelengths (3λ≈21 nm). In this case, the maximum magnitude of velocity is 62.8 m/s (Figure 3a). Nevertheless, when the thermal noise is completely suppressed in the coherent acoustic phonons ($C_{th}=0$), the de Broglie wavelength of the Li ion is still longer than the Li atomic distance (0.6 nm), as shown in Figure 3b. In other words, some quantum effect could still occur at 1 THz under the condition. However, when the thermal noise is suppressed to 6% ($C_{th}=0.06$) in this case, the de Broglie wavelength becomes shorter than the Li atomic distance (0.6 nm) for most of the acoustic period, as shown in Figure 3c. The thermal noise should be suppressed to less than about 3% ($C_{th}\leq 0.03$) in order for the de Broglie wavelength to be longer than the Li atomic distance (0.6 nm) during the whole acoustic period. When $0.03<C_{th}\leq 0.07$, the de Broglie wavelength becomes longer than the Li atomic distance for only a fraction of the acoustic period, as shown in Figure 3c. Thus, in the present case, the superconductivity (superfluidity) of ^6^Li^+^ ions (bosons) could be expected when the thermal noise is suppressed to less than about 3% (or at least less than about 7%).

Experimentally, the thermal noise in coherent phonons could possibly be monitored by a femtosecond probe laser using machine learning to subtract the electronic measurement noise [52,53,54].

Finally, mechanisms for the generation of coherent acoustic phonons are discussed. For repetitive pulsed-laser irradiation, coherent phonons are generated by the displacement of ions in the solid by the electromagnetic field of the laser [55]. This can be expressed by the following equation:

(10) $$\rho \frac{\partial^{2}u}{\partial t^{2}}=-\rho \omega_{0}^{2}u-B\frac{dE_{g}}{dp}\frac{\partial \left(\delta n_{e}\right)}{\partial x}$$

where ρ is the density of the isotropic solid crystal, u is the local displacement of the crystal lattice in the direction of phonon propagation (x direction), $\omega_{0}$ is the angular frequency of the phonon mode, B is the bulk modulus, $E_{g}$ is the band gap, p is pressure, and $\delta n_{e}$ is the change in the electron number density (in a semiconductor) by laser-light irradiation [42]. Equation (10) is equivalent to Equation (11):

(11) $$\frac{\partial^{2}Q}{\partial t^{2}}+\omega_{0}^{2}Q=f\left(\delta n_{e}\right)$$

where Q is the coherent phonon amplitude [56], and $f\left(\delta n_{e}\right)$ is a function of $\delta n_{e}$. Equation (11) means that the phonon mode is excited by an abrupt change in the electron number density ($\delta n_{e}$) by laser-light irradiation. The resultant number of coherent phonons is quite large [56]. In other words, there appears to be a macroscopically large number of phonons in a single quantum state (with a large occupation number). The repetitive excitation of coherent phonons by repetitive pulsed-laser irradiation results in almost no thermal noise because each excitation decays immediately before any thermalization. Accordingly, very low values of $C_{th}$ assumed in the present study would be achievable.

With regard to the possible generation of coherent phonons by ultrasound irradiation using a transducer, the resonance frequency of the system with a relatively high Q (quality) factor may be used because in such a system, acoustic waves of other frequencies are immediately damped out [57,58,59]. However, the amplitude of coherent phonons should not be too large to neglect nonlinear effects.

The aim of the present paper is to present a new idea for the superconductivity of ^6^Li^+^ ions in solid electrolytes under coherent acoustic phonons. The Gross–Pitaevskii equation may not be suitable for the present system due to strong ^6^Li^+^ ion–ion interactions in contrast to the case of the Bose–Einstein condensation of dilute alkali gases [27]. The Ginzburg–Landau theory could be applied to the present system in order to study its stability, which will be a future task.

## 4. Conclusions

It is numerically shown that the de Broglie wavelength of Li ions could be longer than the Li atomic distance in solid electrolytes at room temperature under coherent acoustic phonons when thermal noise is sufficiently suppressed by them. This suggests that the superconductivity of ^6^Li^+^ ions (bosons) could occur under these conditions. In a mixture of ^6^Li^+^ and ^7^Li^+^ ions, only a fraction of the solid could undergo superconductivity. A lower frequency of coherent acoustic phonons is better for this possibility.

## Data Availability

Data are contained within the article.

## References

[1] Mantiram A., Yu X., Wang S. (2017). Lithium battery chemistries enabled by solid-state electrolytes. Nat. Rev. Mater..

[2] Yasui K., Hamamoto K. (2024). Soft matter electrolytes: Mechanism of ionic conduction compared to liquid or solid electrolytes. Materials.

[3] Kanamura K. (2021). Next Generation Batteries.

[4] Randau S., Weber D.A., Kötz O., Koerver R., Braun R., Weber A., Ivers-Tiffée E., Adermann T., Kulisch J., Zeier W.G. (2020). Benchmarking the performance of all-slid-state lithium batteries. Nat. Energy.

[5] Janek J., Zeier W.G. (2016). A solid future for battery development. Nat. Energy.

[6] Yasui K., Hamamoto K. (2023). Influence of dislocations on ionic conductivity and dendrite formation in solid electrolytes. Phys. Scr..

[7] Yasui K., Hamamoto K. (2024). Possibility of high ionic conductivity and high fracture toughness in all-dislocation-ceramics. Materials.

[8] Yasui K. (2024). Merits and demerits of machine learning of ferroelectric, flexoelectric, and electrolytic properties of ceramic materials. Materials.

[9] Yasui K., Hamamoto K. (2024). Toward all-dislocation-ceramics for high ionic conductivity produced by dry pressing at relatively low temperatures with and without ultrasound. J. Appl. Phys..

[10] Owens B.B., Argue G.R. (1967). High-conductivity solid electrolytes: $M{Ag}_{4}I_{5}$. Science.

[11] Muy S., Schlem R., Shao-Horn Y., Zeier W.G. (2021). Phonon-ion interactions: Designing ion mobility based on lattice dynamics. Adv. Energy Mater..

[12] Li Y., Song S., Kim H., Nomoto K., Kim H., Sun X., Hori S., Suzuki K., Matsui N., Hirayama M. (2023). A lithium superionic conductor for millimeter-thick battery electrode. Science.

[13] Fuchs T., Culver S.P., Till P., Zeier W.G. (2020). Defect-mediated conductivity enhancements in ${Na}_{3-x}{Pn}_{1-x}W_{x}S_{4}$ (Pn = P, Sb) using aliovalent substitutions. ACS Energy Lett..

[14] Ling C. (2023). Phonon-promoted superionic conduction in fluorite-structured compounds. Chem.

[15] He X., Zhu Y., Mo Y. (2017). Origin of fast ion diffusion in super-ionic conductors. Nat. Commun..

[16] Wakamura K. (1997). Roles of phonon amplitude and low-energy optical phonons on superionic conduction. Phys. Rev. B.

[17] Rice M.J., Roth W.L. (1972). Ionic transport in super ionic conductors: A theoretical model. J. Solid. State Chem..

[18] Schrieffer J.R. (2018). Theory of Superconductivity.

[19] Bardeen J., Cooper L.N., Schrieffer J.R. (1957). Theory of superconductivity. Phys. Rev..

[20] Combescot R. (2022). Superconductivity: An Introduction.

[21] Bednorz J.G., Müller K.A. (1986). Possible high $T_{c}$ superconductivity in the Ba-La-Cu-O system. Z. Phys. B.

[22] Schrieffer J.R., Brooks J.S. (2007). Handbook of High-Temperature Superconductivity: Theory and Experiment.

[23] Takeshita N., Yamamoto A., Iyo A., Eisaki H. (2013). Zero resistivity above 150 K in $Hg{Ba}_{2}{Ca}_{2}{Cu}_{3}O_{8+\delta }$ at high pressure. J. Phys. Soc. Jpn..

[24] Yamamoto A., Takeshita N., Terakura C., Tokura Y. (2015). High pressure effects revisited for the cuprate superconductor family with highest critical temperature. Nat. Commun..

[25] Drozdov A.P., Kong P.P., Minkov V.S., Besedin S.P., Kuzovnikov M.A., Mozaffari S., Balicas L., Balakirev F.F., Graf D.E., Prakapenka V.B. (2019). Superconductivity at 250 K in lanthanum hydride under high pressures. Nature.

[26] Somayazulu M., Ahart M., Mishra A.K., Geballe Z.M., Baldini M., Meng Y., Struzhkin V.V., Hemley R.J. (2019). Evidence for superconductivity above 260 K in lanthanum superhydride at megabar pressures. Phys. Rev. Lett..

[27] Leggett A.J. (2006). Quantum Liquids, Bose Condensation and Cooper Pairing in Condensed-Matter Systems.

[28] Kittel C., Kromer H. (1980). Thermal Physics.

[29] Landau L.D., Lifshitz E.M. (1980). Statistical Physics.

[30] Hamao N., Yamaguchi Y., Hamamoto K. (2021). Densification of a NASICON-type LATP electrolyte sheet by a cold-sintering process. Materials.

[31] Dai P., Liu Y., Yi S., Zhou S., Liu Y., Gao T., Cao G., Zhao S. (2024). Cold-sintering assisted process enables densified and robust fine-grained ${Li}_{1.3}{Al}_{0.3}{Ti}_{1.7}{\left(PO_{4}\right)}_{3}$ electrolytes for solid-state batteries. J. Power Sources.

[32] Monchak M., Hupfer T., Senyshyn A., Boysen H., Chernyshov D., Hansen T., Schell K.G., Bucharsky E.C., Hoffmann M.J., Ehrenberg H. (2016). Lithium diffusion pathway in ${Li}_{1.3}{Al}_{0.3}{Ti}_{1.7}{\left(PO_{4}\right)}_{3}$ (LATP) superionic conductor. Inorg. Chem..

[33] Zhang Z.-W., Pethick C.J. (2021). Proton superconductivity in pasta phases in neutron star crusts. Phys. Rev. C.

[34] Sedrakian D.M., Sedrakian A.D., Zharkov G.F. (1997). Type I superconductivity of protons in neutron stars. Mon. Not. R. Astron. Soc..

[35] Tuoriniemi J., Juntunen-Nurmilaukas K., Uusvuori J., Pentti E., Salmela A., Sebedash A. (2007). Superconductivity in lithium below 0.4 millikelvin at ambient pressure. Nature.

[36] Atkins P.W. (1982). Physical Chemistry.

[37] Misochko O.V. (2001). Coherent phonons and their properties. J. Exp. Theor. Phys..

[38] Ventura-Velázquez C., Ávila B.J., Kyoseva E., Rodríguez-Lara B.M. (2019). Robust optomechanical state transfer under composite phase driving. Sci. Rep..

[39] Misochko O.V., Hase M., Ishioka K., Kitajima M. (2004). Transient Bose-Einstein condensation of phonons. Phys. Lett. A.

[40] Garrett G.A., Rojo G., Sood A.K., Whitaker J.F., Merlin R. (1997). Vacuum squeezing of solids: Macroscopic quantum states driven by light pulses. Science.

[41] Ruello P., Gusev V.E. (2015). Physical mechanisms of coherent acoustic phonons generation by ultrafast laser action. Ultrasonics.

[42] Thomsen C., Grahn H.T., Maris H.J., Tauc J. (1986). Surface generation and detection of phonons by picosecond light pulses. Phys. Rev. B.

[43] Kittel C. (2005). Introduction to Solid State Physics.

[44] McQuarrie D.A., Simon J.D. (1997). Physical Chemistry: A Molecular Approach.

[45] Murray É.D., Fritz D.M., Wahlstrand J.K., Fahy S., Reis D.A. (2005). Effect of lattice anharmonicity on high-amplitude phonon dynamics in photoexcited bismuth. Phys. Rev. B.

[46] Andreev A.F., Lifshitz I.M. (1969). Quantum theory of defects in crystals. Sov. Phys. JETP.

[47] Galli D.E., Reatto L. (2001). Vacancies in solid ^4^He and Bose Einstein condensation. J. Low Temp. Phys..

[48] Burns C.A., Goodkind J.M. (1994). The role of vacancies in solid ^4^He. Phys. B.

[49] Leggett A.J. (1970). Can a solid be “superfluid”?. Phys. Rev. Lett..

[50] Gautam S.K., Singh F., Sulania I., Singh R.G., Kulriya P.K., Pippel E. (2014). Micro-Raman study on the softening and stiffening of phonons in rutile titanium dioxide film: Competing effects of structural defects, crystallite size, and lattice strain. J. Appl. Phys..

[51] Ferrier-Barbut I., Delehaye M., Laurent S., Grier A.T., Pierce M., Rem B.S., Chevy F., Salomon C. (2014). A mixture of Bose and Fermi superfluids. Science.

[52] Wright O.B., Kawashima K. (1992). Coherent phonon detection from ultrafast surface vibrations. Phys. Rev. Lett..

[53] Makhrinov V., Maksut Z., Yazici A., Almagambetov A., Grossan B., Shafiee M. (2025). Superconducting microresonator signal denoising using machine learning. IEEE Trans. Appl. Supercond..

[54] Pratticò D., Laganà F., Oliva G., Fiorillo A.S., Pullano S.A., Calcagno S., De Carlo D., Foresta F.L. (2025). Integration of LSTM and U-net models for monitoring electrical absorption with a system of sensors and electronic circuits. IEEE Trans. Instrum. Meas..

[55] Cheng T.K., Vidal J., Zeiger H.J., Dresselhaus G., Ippen E.P. (1991). Mechanism for displacive excitation of coherent phonons in Sb, Bi, Te, and ${Ti}_{2}O_{3}$. Appl. Phys. Lett..

[56] Kuznetsov A.V., Stanton C.J. (1994). Theory of coherent phonon oscillations in semiconductors. Phys. Rev. Lett..

[57] Kinsler L.E., Frey A.R., Coppens A.B., Sanders J.V. (1982). Fundamentals of Acoustics.

[58] Kuttruff H. (2007). Acoustics, an Introduction.

[59] Yasui K. (2018). Acoustic Cavitation and Bubble Dynamics.

